# The SquAd derivation: A Square Additive approach to the turbulent Prandtl number

**DOI:** 10.12688/openreseurope.15367.1

**Published:** 2023-03-07

**Authors:** Vincent Moreau

**Affiliations:** 1 CRS4, Science and Technology Park, Piscina Manna, 09050, Cagliari, Italy

**Keywords:** Prandtl, Kays, CFD, turbulence modelling

## Abstract

Liquid metals have been chosen as primary coolant of innovative nuclear systems under current development. They present a very high thermal conductivity and hence a very low molecular Prandtl number. This feature challenges the modeling of turbulent thermal flows applying the Reynolds analogy. This paper addresses this challenge. A new formula for the turbulent Prandtl number is derived in terms of local variables available from two-equations turbulence models. The derivation is a direct consequence of the expected square additivity of the molecular and turbulent parameters defining the effective viscosity and the effective conductivity. The formula does not degenerate and leads to a Kays like formulation if approximated. While constrained by the quality of the turbulent viscosity modeling, it has the potential to improve the numerical simulation of turbulent thermal flows.

## Introduction

Low Prandtl number liquid metals serve as primary coolant for MYRRHA ^1^ and ALFRED ^2^, two Gen-IV reactors under development ^1^. The low Prandtl number induces discrepancies in the modeling of the turbulent heat transfer when directly addressed according to the Reynolds analogy with a constant turbulent Prandtl number. The thermal boundary layer is considerably larger than the velocity one, up to the point that, while we can clearly define a bulk velocity, the temperature profile may not exhibit any almost constant bulk temperature plateau. The issue was investigated by several authors who reviewed the existing correlations and proposed their own one ^2– 4^. The correlations make use of adimensional numbers such as Reynolds and Peclet. Their functional form is mostly empirical with coefficients determined by best fit. The Reynolds number and the Peclet numbers are global parameters, only useful and well-defined for simple geometries. Their use in CFD ^3^ simulations of complex geometries is questionable. Among all correlations reviewed, Kays’ correlation is the only one making exclusive use of local parameters. Variants of this correlation have been used with significant success. The variants share the same structure but feature different values for one numerical constant. In our previously published four page brief report ^5^, we showed that the correlations can be simply derived on a basic assumption with regards to the non-linear combination of stochastic effects and the variants then stem from different approximations of a mother formula. The objective of this former brief report was only to establish and keep trace of the probable relationship between the stochastic concept and the empirical correlation. It is in no way a bulletproof demonstration and there is no specific treatment of the viscosity. In particular, the definition of the asymptotic Prandtl number and its use are not completely consistent. Besides, from the purely numerical point of view, a defect of the mother formula, which is transferred to the variants, is that the turbulent Prandtl number becomes infinite at vanishing turbulence.

These considerations motivate to proceed further with the analysis which is the object of this current brief report. The key point resides in refining the concept that lies behind the formerly loosely determined asymptotic Prandtl number. What was really needed to be done was apparently to clearly separate what is from molecular origin and what is from turbulent origin. This was found to be more prolifically reformulated in terms of differentiating the properties of the fluid from the properties of the flow, being this later one either laminar or turbulent.

In order to proceed consistently with the flow versus fluid properties separation it becomes necessary to apply the principle of square additivity not only to the thermal conductivity but also to the viscosity. This allows us to have a cleaned definition of what is now called the ”flow Prandtl number” which is conceived to be a universal constant of the flow, independent of the fluid. As previously, the principle of square additivity completely determines the turbulent Prandtl number. The new formula, while similar, is more articulated than the previous one presented. Its first order approximation still has the functional shape of Kay’s correlation. It however gives better insight on its probable range of validity. Besides, as a good news for numerical implementation, the newly derived formula has the merit of not degenerating anymore at vanishing turbulence, something which was not a feature of the previous formula.

## Turbulent prandtl number derivation

In the the framework of thermal fluid dynamics of turbulent flows, the focus is concentrated on the determination and modelling of the effective viscosity and effective heat diffusion of the fluid. The viscosity and the heat diffusion both consist in the sum of two parts, one molecular and the other turbulent. This is a convenient and simple representation based on the fact that for most turbulent flows, the turbulent viscosity and conductivity are much higher than their molecular counterparts. The turbulent quantities were also not expected, at least historically, to be known at a high level of precision.

With regard to the heat diffusion, both the molecular and the turbulent fluxes are oriented in the direction of the local temperature gradient and proportional to it. The intensity is also determined by conductivity coefficients. The effective conductivity coefficient *k _e_* can be expressed as


$$\;k_{e}=k+k_{t}\;(1)$$

denoting, respectively with *k* and *k _t_* the molecular conductivity and the increment due to turbulence.

Indicating with *ρ* the fluid density and *C _p_* its specific heat, this expression can be rewritten in terms of thermal diffusivity:


$$\;\alpha_{e}=\alpha +\alpha_{t}\;(2)$$

in which *α _e_* = *k _e_*/( *ρC _p_*) stands for the effective diffusivity, *α* = *k*/( *ρC _p_*) is the molecular part and *α _t_* = *k _t_*/( *ρC _p_*) is the turbulent part, *ρ* is the density and *C _p_* the specific heat.

Similarly, as far as the viscosity in concerned, the effective kinematic viscosity *ν _e_* is the sum of a laminar contribution *ν* and a turbulent contribution *ν _t_*:


$$\;\nu_{e}=\nu +\nu_{t}\;(3)$$

The argument previously developed in 5 is the following. Conduction has a stochastic origin. The scale at which molecular and turbulent conduction operate are different and their mechanisms are unrelated. Molecular conduction is a molecular process and is a property of the fluid while turbulent conduction has a convective origin and is a property of the flow. The combination of their effects is better represented as a convolution rather than as a direct sum. Translated in formula, we expect to have:


$$\;\alpha_{e}=\sqrt{\alpha^{2}+\alpha_{0}^{2}},\;(4)$$

where the turbulent diffusivity *α*_0_ should be an intrinsic property of the flow rather than the fluid.

The same argument is extended to the viscous process and states that the effective viscosity comes from two independent processes whose intensity should be square additive:


$$\;\nu_{e}=\sqrt{\nu^{2}+\nu_{0}^{2}}.\;(5)$$

By simple substitution, we have just defined two quantities:


$$\;\nu_{0}=\nu_{t}\sqrt{1+\frac{2\nu }{\nu_{t}}}\;(6)$$

and


$$\alpha_{0}=\alpha_{t}\sqrt{1+\frac{2\alpha }{\alpha_{t}}}.\;(7)$$

We can also redefine *ν _t_* and *α _t_* in terms of these quantities:


$$\nu_{t}=\sqrt{\nu^{2}+\nu_{0}^{2}}-\nu \;(8)$$

$$\alpha_{t}=\sqrt{\alpha^{2}+\alpha_{0}^{2}}-\alpha \;(9)$$

We now introduce the Prandlt number $Pr=\frac{\nu }{\alpha }$ , the turbulent Prandtl number $Pr_{t}=\frac{\nu_{t}}{\alpha_{t}}$ and the flow Prandtl number as $Pr_{0}=\frac{\nu_{0}}{\alpha_{0}}$ . This later number has been conceived in order to be independent of the fluid and only dependent on the flow. Noting that $\frac{\alpha }{\alpha_{0}}=\frac{Pr_{0}\nu }{Pr\nu_{0}}$ , by combining the former equations, we obtain:


$$Pr_{t}=\frac{Pr_{0}^{2}}{Pr}\frac{\sqrt{1+{\left(\frac{Pr\nu_{0}}{Pr_{0}\nu }\right)}^{2}}+1}{\sqrt{1+{\left(\frac{\nu_{0}}{\nu }\right)}^{2}}+1}.\;(10)$$

This specific form is chosen to show that it cannot degenerate to zero or infinity. In effect, because *ν*_0_ goes to zero if *ν _t_* does, then *Pr _t_* tends to $\frac{Pr_{0}^{2}}{Pr}$ while for large turbulence, *Pr _t_* tends to *Pr*_0_. In turn, if *Pr*_0_, built for this purpose, does not degenerate, then neither does *Pr _t_*.

The former expression can be rewritten in terms of *ν*/ *ν*_0_:


$$Pr_{t}=Pr_{0}\frac{\sqrt{1+{\left(\frac{Pr_{0}\nu }{Pr\nu_{0}}\right)}^{2}}+\frac{Pr_{0}\nu }{Pr\nu_{0}}}{\sqrt{1+{\left(\frac{\nu }{\nu_{0}}\right)}^{2}}+\frac{\nu }{\nu_{0}}}.\;(11)$$

This formula is quite complicated and not easy to interpret at first glance, but considering *ν*/ *ν*_0_ as a small parameter, a brutal simplification of 11 at first order in this parameter gives:


$$Pr_{t}≃Pr_{0}[1+(\frac{Pr_{0}}{Pr}-1)\frac{\nu }{\nu_{0}}]\;(12)$$

This formulation is practical only if *ν*_0_ is readily available. This would be the case if we had a transport equation for *ν*_0_ or a related variable similarly to what is done with the usual 2-equations turbulence models. Exploring the potential of this possibility is beyond the scope of the current argument and we need to express *Pr _t_* in terms of known parameters.

Thus, expressing *Pr _t_* in terms of *ν _t_* instead of *ν*_0_, the formula becomes:


$$Pr_{t}=\frac{Pr_{0}^{2}}{Pr}\frac{\sqrt{1+{\left(\frac{Pr}{Pr_{0}}\right)}^{2}\frac{\nu_{t}}{\nu }\left(\frac{\nu_{t}}{\nu }+2\right)}+1}{2+\frac{\nu_{t}}{\nu }},\;(13)$$

this form being useful for interpretation at vanishing turbulent viscosity.

Dividing this last formula by *Pr*_0_, we have a relation between the three adimentional groups $\frac{Pr_{t}}{Pr_{0}}$ , $\frac{Pr}{Pr_{0}}$ and $\frac{\nu_{t}}{\nu }$ in the form:


$$\frac{Pr_{t}}{Pr_{0}}=f(\frac{Pr}{Pr_{0}},\;\frac{\nu_{t}}{\nu }).\;(14)$$

In view of a development in *ν*/ *ν _t_*, the formula becomes:


$$Pr_{t}=Pr_{0}\frac{\sqrt{1+\frac{2\nu }{\nu_{t}}+{\left(\frac{Pr_{0}\nu }{Pr\nu_{t}}\right)}^{2})}+\frac{Pr_{0}\nu }{Pr\nu_{t}}}{1+\frac{2\nu }{\nu_{t}}}\;(15)$$

Here again, a brutal first order development in terms of *ν*/ *ν _t_*, valid only when both *ν _t_*/ *ν* and *Prν _t_*/ *Pr*_0_ *ν* are large, gives:


$$Pr_{t}≃Pr_{0}[1+(\frac{Pr_{0}}{Pr}-1)\frac{\nu }{\nu_{t}}],\;(16)$$

meaning that changing from *ν*_0_ to *ν _t_* brings only second order terms approximation. In particular, this last expression has the same form as the Kays correlation ^2^.

## Discussion

Under the hypothesis that the flow Prandtl number *Pr*_0_ is constant, it coincides with the value used for highly turbulent flow and near unit *Pr*. That is, *Pr*_0_ = 0.85. We get for *Pr* ≃ 0.025 typical of heavy liquid metal:


$$Pr_{t}≃0.85+\frac{0.70}{Pr\frac{\nu_{t}}{\nu }}\;(17)$$

For the second coefficient (here 0.7), Kays indicated two values, 0.7 and 2, discussing without reaching a conclusion in favor of one or the other value. However, a less brutal approximation could lead to a different coefficient. For example, in 6, 1.46 is obtained from direct analytical integration and best fit of heat transfer in a tube. Moreover, our newly derived formula is not much different than the one derived previously ^5^ and a more precise approximation, while more complex to derive, would most probably also lead to an increased second coefficient about 1.45.

There are indications ^2^ that *Pr _t_* and therefore also *Pr*_0_ is about 0.85 for very large *ν _t_*/ *ν*. We do not know the behavior of *Pr*_0_ for lower values. Nevertheless, all this construction makes sense only under the hypothesis of a basically constant *Pr*_0_. A particular case is when *Pr* = *Pr*_0_. Then *Pr _t_* = *Pr*_0_ too. For medium and high *Pr*, we observe that *Pr _t_* in formula ( 11) does not significantly departs from *Pr*_0_, except for strongly vanishing *ν _t_*/ *ν*.

With a little algebra, we found that *Pr _t_* is a decreasing function of *ν _t_* for *Pr* ≤ *Pr*_0_ and increasing function of *ν _t_* for *Pr* ≥ *Pr*_0_, with values spanning the interval [ *Pr*_0_; $Pr_{0}^{2}$ / *Pr*] and [ $Pr_{0}^{2}$ / *Pr*; *Pr*_0_]. The approximation 16, while with the same monotonicity, fails to meet the correct bound for vanishing viscosity, for which it degenerates. Interestingly, we can see that the second coefficient depends critically on the Prandtl number, to the point that it vanishes for *Pr _t_* = *Pr*_0_ and changes sign afterwards, like for the complete expression. This is a clear indication that the Kays correlation should be used as such only for low Prandtl number (say < 0.1) fluids.

The derived formula is ought to be used within a turbulence model. It would make sense only if the turbulence model correctly predicts the turbulent viscosity. This has to be true not only in the viscous boundary layer but also and principally in the bulk, in which the thermal boundary layer could still be in development. The problem is that the turbulence models mainly focus on the correct near-wall boundary layer turbulent viscosity profile, as it is the place where almost all the pressure drop is built, with the main aim to capture the correct wall shear stress. The turbulent viscosity profile in the bulk is normally of no practical importance, except for thermal flows of low Prandtl fluids. Looking at the profile of *ν _t_* compared with direct numerical simulation data even in the simpliest 2D channel flow, see figure 5 in 7, we can see that the turbulence models fail to give a correct profile for a wall Y+ above 30. In particular, *ν _t_* is underpredicted below Y+ up to 100-150 and over predicted afterward. The difference between the simply additive and the square additive approaches is mainly concentrated and felt around the values where both contributions are of similar intensity: *ν*/ *Pr* ∽ *ν _t_*/ *Pr*_0_ or equivalently *ν _t_*/ *ν* ∽ *Pr*_0_/ *Pr*. For a low Prandtl fluids with *Pr* = 0.025 we have *Pr*_0_/ *Pr* = 34, so all the region where *ν _t_*/ *ν* lies between say 10 and 100 is concerned, that is precisely for Y+ above 30 for the case analysed in 7. The balance between the thermal effects of both Y+ region is shifted towards an artificially increased diffusion. To counteract this effect, the turbulent Prandtl number can be increased artificially for a better fit. This could be an explanation for the use of an augmented second coefficient in the Kays correlation as indicated previously.

The main effect of applying the square additivity to the effective viscosity is that it removes the degeneration of the *Pr _t_* formula for vanishing viscosity that was still present in 5. It is not clear nor sure that it is of practical importance anywhere else. It seems that, within the level of approximation given by the 2-equations turbulence models, *ν _t_* and *ν*_0_ can be used indifferently in the formula. In other words, the square additivity could be used solely for the energy equation.

## Conclusions

We consider that turbulent viscosity and molecular viscosity are originated from two independent stochastic processes whose intensity is square additive. We make the same consideration for the turbulent conductivity and the thermal conductivity. We have defined a flow Prandtl number which is expected to be a universal property of the flow and to be in fact a constant under the square additive approach. The Prandtl number is then determined by a formula reproducing the first variant of Kays’ correlation when approximated at the first order. The derivation sheds light on the Kays correlation and indicates that the second coefficient depends critically on the Prandtl number to the point that it vanishes when *Pr* = 0.85. Under the condition that the classical 2-equations turbulence models become able to capture correctly the turbulent viscosity profile, we expect that directly implementing the square additivity of the components of the effective conductivity could give improved thermal results independently of the Prandtl number and particularly for the low Prandtl liquid Lead and Lead alloys.

The Reynolds analogy and its extension to thermal flows could have a much wider domain of validity than expected by combining it with the square additivity of the coefficients.

## Data Availability

No data are associated with this article.

## Footnotes

^1^ Multi-purpose Hybrid Research Reactor for High-tech Applications

^2^ Advanced Lead-cooled Fast Reactor European Demonstrator

^3^ Computational Fluid Dynamics
