# Supplementary material for: The SquAd derivation: A Square Additive approach to the turbulent Prandtl number
Source: Open Res Eur. 2023 Sep 13;3:43. Originally published 2023 Mar 7. [Version 3] doi: 10.12688/openreseurope.15367.3 (PMC10873542; doi:10.12688/openreseurope.15367.3)
Supplement: Peer review history

# Reviewer report: Reviewer response for version 3

**DOI:** 10.21956/openreseurope.17933.r35103

**Published:** 2024-02-26

**Authors:** Davide Modesti

**Affiliations:** 1 Delft University of Technology, Mekelweg, Delft, The Netherlands

The major issues that I pointed out from the first revision have not been addressed, therefore I cannot recommend the manuscript for indexing. I summarize again the main issues. The author proposes a new definition of effective viscosity and effective thermal diffusivity, namely \alpha_e = sqrt(\alpha^2 + \alpha_0^2), where \alpha_0 is a property of the fluid. This new definition is used to derive a new formula for the turbulent Prandtl number

**Major issues:** 1) The new definitions (equations 4 and 5) don't have a clear theoretical fundation, or if they do it is not clear. The canonical definition of effective thermal diffusivity stems from the mean thermal balance, when using \overline{T'v'} = \alpha_T dT/dy. What is instead the reasoning behind equations (4) and (5). For instance, why using this definition and not \alpha_e = (\alpha^4 + \alpha_0^4)^(1/4), or any other exponent? Why not another function f(alpha,alpha_0)? 2) The author did not validate his hypothesis with experimental or numerical data, therefore I cannot comment on the accuracy of the validity of the hypothesis, as the paper does not contain any figure. I would like to see a comparison of the proposed turbulent Prandtl number with the Prandtl number computed from available DNS or experiments. DNS data at low Prandtl number are available, see for instance the work by Pirozzoli, Sergio. "An explicit representation for mean profiles and fluxes in forced passive scalar convection." Journal of Fluid Mechanics 968 (2023): R1. In the previous paper by Pirozzoli, the eddy diffusivity is found to be nearly independent from the Prandtl number, which seems to contradict the main point of the author.

Is the study design appropriate and does the work have academic merit?

Partly

Is the work clearly and accurately presented and does it cite the current literature?

Partly

If applicable, is the statistical analysis and its interpretation appropriate?

Not applicable

Are all the source data underlying the results available to ensure full reproducibility?

No source data required

Are the conclusions drawn adequately supported by the results?

No

Are sufficient details of methods and analysis provided to allow replication by others?

Yes

Reviewer Expertise:

Wall turbulence; turbulent flows; compressible flows; forced thermal convection; heat transfer

I confirm that I have read this submission and believe that I have an appropriate level of expertise to state that I do not consider it to be of an acceptable scientific standard, for reasons outlined above.

# Reviewer report: Reviewer response for version 3

**DOI:** 10.21956/openreseurope.17933.r37496

**Published:** 2024-02-16

**Authors:** Agustín Villa Ortiz

**Affiliations:** 1 Universidad Rey Juan Carlos, Madrid, Spain

This work is a nice derivation of a Prt formula that could be used later in thermal flows.

 I have a comment on the introduction of the turbulent Prandtl number, Reynolds analogy... The Simple Gradient Diffusion Hypothesis (SGDH) should be mentioned anywhere before the text:

*"With regard to the heat diffusion, both the molecular and the turbulent fluxes are oriented in the direction of the local temperature gradient and they are proportional to it. The intensity is also determined by the conductivity coefficients."*

 In the case of turbulent heat fluxes (THF) it is not possible to assume that in general both THF and temperature gradient vectors are proportional (please check natural convection cases). If you assume the SGDH and therefore use the Reynolds Analogy you can for sure affirm your comment.

 A few figures -where you show the similarities between SquAd and Kays, or the range of validity your simplifications (formulas 10 to 11 and 15 to 16) on the ν/ν _0_ ν/ν _t_... ratios- might help to validate your approach. Even the comparison of SquAd and Kays for a simple 2D flow at different Prandtl numbers should demonstrate the SquAd effectiveness and preference over Reynolds Analogy.

Is the study design appropriate and does the work have academic merit?

Yes

Is the work clearly and accurately presented and does it cite the current literature?

Yes

If applicable, is the statistical analysis and its interpretation appropriate?

Not applicable

Are all the source data underlying the results available to ensure full reproducibility?

No source data required

Are the conclusions drawn adequately supported by the results?

Partly

Are sufficient details of methods and analysis provided to allow replication by others?

Partly

Reviewer Expertise:

Liquid metals, RANS themal turbulence modeling

I confirm that I have read this submission and believe that I have an appropriate level of expertise to confirm that it is of an acceptable scientific standard, however I have significant reservations, as outlined above.

# Reviewer report: Reviewer response for version 3

**DOI:** 10.21956/openreseurope.17933.r37511

**Published:** 2024-02-16

**Authors:** Andrea Pucciarelli

**Affiliations:** 1 University of Pisa, Pisa, Tuscany, Italy

The contribution proposes an interesting approach for the calculation of the effective viscosity (and diffusivity) by means of a square additive approach.

 The hypothesis leads to a formulation of a relation allowing the calculation of the Prtur which is qualitatively similar to Kays relation.

 In this regard, in the Kays equation at the denominator there is a Peclet number. In Equation 17 the role of the Reynolds number is made by Nit/Ni. While I understand that the higher the Reynolds the higher (probably) the turbulent viscosity, it would be nice understanding or commenting how these two quantities scale and if there is any limitation in this sense to its application. 

 Concerning the approval of this contribution, it depends on the spirit of this repository.

 As a CFD user I would like to have an idea of the applicability of the present approach to calculations; it is also to be understood how it copes with the eddy viscosity field relations proposed in literature. A validation in this sense would be required for indexing. I do understand it is not the aim of this report, which clearly states that "It is not clear nor sure that it is of practical importance anywhere else" except for the present application.

 Nevertheless, I do believe it is worth sharing this idea, the obtained similarity is for sure fascinating, it got my curiosity and I think I could give it a try in some calculations of ours.

 Thank you for sharing the contribution.

Is the study design appropriate and does the work have academic merit?

Partly

Is the work clearly and accurately presented and does it cite the current literature?

Partly

If applicable, is the statistical analysis and its interpretation appropriate?

Not applicable

Are all the source data underlying the results available to ensure full reproducibility?

No source data required

Are the conclusions drawn adequately supported by the results?

Partly

Are sufficient details of methods and analysis provided to allow replication by others?

Partly

Reviewer Expertise:

Computational Fluid Dynamics

I confirm that I have read this submission and believe that I have an appropriate level of expertise to confirm that it is of an acceptable scientific standard, however I have significant reservations, as outlined above.

# Reviewer report: Reviewer response for version 2

**DOI:** 10.21956/openreseurope.17529.r33956

**Published:** 2023-08-22

**Authors:** Iztok Tiselj

**Affiliations:** 1 Faculty of Mathematics and Physics, Jožef Stefan Institute, Ljubljana, Slovenia

I share the opinion of the other reviewers:

1. This is an interesting approach to modelling of turbulent Prandtl number. However, it is not completely clear why replacement of one type of flow-dependent and material-independent variables (turbulent viscosity and turbulent diffusivity) with another pair of similar variables is really beneficial.
2. The main concern: the advantage of a new approach should be validated with available numerical data and experiments. The future and further applications of the proposed approach will show, whether the added value is relevant.

Is the study design appropriate and does the work have academic merit?

Partly

Is the work clearly and accurately presented and does it cite the current literature?

Partly

If applicable, is the statistical analysis and its interpretation appropriate?

Not applicable

Are all the source data underlying the results available to ensure full reproducibility?

No source data required

Are the conclusions drawn adequately supported by the results?

Partly

Are sufficient details of methods and analysis provided to allow replication by others?

Yes

Reviewer Expertise:

References relevant for the present review: DNS simulations of channel flow heat transfer with and without conjugate heat transfer at low Prandtl numbers.

I confirm that I have read this submission and believe that I have an appropriate level of expertise to confirm that it is of an acceptable scientific standard, however I have significant reservations, as outlined above.

# Response

**Published:** 2023-09-06

**Authors:** Vincent MOREAU

**Affiliations:** BCISP-QC, CRS4, Pula (CA), Italy

The reviewer text is indicated in slanted. *I share the opinion of the other reviewers:*

*This is an interesting approach to modelling of turbulent Prandtl number. However, it is not completely clear why replacement of one type of flow-dependent and material-independent variables (turbulent viscosity and turbulent diffusivity) with another pair of similar variables is really beneficial.* I think that this paper is bringing undue expectations.

 No replacement is required. Enforcing the equivalence of two different approaches to the same phenomena had the result to produce automatically a formula for the turbulent Prandtl number. The formula is substantially equivalent to Kays correlation for liquid metals and revert to the classical constant value for near unit Prandtl fluids. That is all, but it is already in my opinion a worthwhile result.

 While the concept was already presented in a previous paper, it was done in an unfinished way and with a confusing terminology. It took me more than a year to realize that there was room for improvement. Systemization of the approach led to the present formula. For this, I had to give first letters then names to things. I understand that giving names to things is quite presumptuous but is has been raised necessary for clarity. 

      

*The main concern: the advantage of a new approach should be validated with available numerical data and experiments. * I strongly disagree with this. The advantage of the new approach is only to give a possible interpretation to Kays correlation and, if one wants, to use a non-degenerating equivalent formula without empirical constant. This is the over-expectation issue. It is not the aim of this paper to validate the Kays correlation with experiments. I chose to make a brief report also to avoid this kind of undue request. Validating Kays correlation is far beyond my competence, budget, time availability and has already been done elsewhere. However, for completeness, and as a compromise, I an adding in the report a more recent publication (ref. 7, 2020) in which Kays correlation is discussed and validated. *The future and further applications of the proposed approach will show, whether the added value is relevant.* For me, re-interpreting an efficient correlation as a formula without empirical constant is already an extremely relevant result. Maybe this is a deformation due to my original formation as a mathematician. I understand though that for most people working in the field, this feature is almost totally irrelevant. I wanted to publish this result for the few others, who can also read simple formulas, if I am let do it. Another point I wanted to stress is the need of a turbulence model giving decent effective viscosity profiles. I have no competency in that matter, so my contribution in any case ends here.

# Reviewer report: Reviewer response for version 2

**DOI:** 10.21956/openreseurope.17529.r33827

**Published:** 2023-08-10

**Authors:** Davide Modesti

**Affiliations:** 1 Delft University of Technology, Mekelweg, Delft, The Netherlands

The manuscript has only slightly changed compared to the original version, and I still have major concerns regarding the validation. For this reason I cannot recommend the article for indexing.

1. Validation cannot be done only with Kays formula. This formula is for Prandtl numbers close to unity and not for low Prandtl numbers. Data from experiments or simulations should be used and figures should be added to the manuscript to support the hypotheses and the results.
2. I still miss how the effective diffusivity can satisfy the mean temperature balance.

Is the study design appropriate and does the work have academic merit?

Partly

Is the work clearly and accurately presented and does it cite the current literature?

Partly

If applicable, is the statistical analysis and its interpretation appropriate?

Not applicable

Are all the source data underlying the results available to ensure full reproducibility?

No source data required

Are the conclusions drawn adequately supported by the results?

No

Are sufficient details of methods and analysis provided to allow replication by others?

Yes

Reviewer Expertise:

Wall turbulence; turbulent flows; compressible flows; forced thermal convection; heat transfer

I confirm that I have read this submission and believe that I have an appropriate level of expertise to state that I do not consider it to be of an acceptable scientific standard, for reasons outlined above.

# Response

**Published:** 2023-09-06

**Authors:** Vincent MOREAU

**Affiliations:** BCISP-QC, CRS4, Pula (CA), Italy

The reviewer text is indicated in slanted. *The manuscript has only slightly changed compared to the original version, and I still have major concerns regarding the validation. For this reason I cannot recommend the article for indexing.* The manuscript has been modified in order to take into account the constructive and relevant indications of the first reviewers. It seems to me that your review is mainly based on misunderstanding. This led me to try to improve the paper clarity.

 One nice thing about the transparency politics of Open-Research-Europe is that in case of disagreement between author and reviewer, an interested reader can make its own informed opinion. *Validation cannot be done only with Kays formula. This formula is for Prandtl numbers close to unity and not for low Prandtl numbers.* First you suggest to use validation from Kays old work and when I stress that this is already done with a much more recent one with all the improvement a researcher can do in 14 years, then you pretend that it is no more valid. This is quite confusing. The affirmation in the second sentence is extremely surprising and I really wonder where it does come from. If you read Kays reference (1994), you will see that this is clearly not the case. Same thing with the more recent reference (2016) from Taler. Maybe you made confusion with eq.21 (Kays and Crawford) in Kays paper related to Prt for air. What is referred as Kays correlation is eq.17 in his 1994 paper (and sometimes also eq.18). *Data from experiments or simulations should be used and figures should be added to the manuscript to support the hypotheses and the results.* I strongly disagree. Kays correlation does not need any more validation. A discussion on this point can be found in a more recent paper:

 Zhang R, Wang Z, Wang Z, Ren T and Shi J (2020) Theoretical Investigation on the Fully Developed Turbulent Heat Transfer Characteristics of Liquid Sodium. Front. Energy Res. 8:10. doi: 10.3389/fenrg.2020.00010

 For completeness, I refer to this paper in the Discussion section and add it in the references. To quote Kays paper: "During the past 40 years dozens of solutions for Prt based on a variety of analytic models have appeared in literature.(...) but virtually all [analyses] contain free constants, which must be evaluated by comparison with the available experimental data."

 My finding is that Kays correlation, which also seems to contain a "free" constant, in fact does not, once you interpret it in the framework of stochastic process.

 In other words, my work is a theoretical validation of Kays correlation and if Kays correlation is wrong or is of very limited range of validity, then the "theory" is also wrong. There is no room and no need in this context for figures. The paper is intended for people who can read simple formulas.  

*I still miss how the effective diffusivity can satisfy the mean temperature balance.* I do not understand what you mean. I do not understand at what level I should state my answer. Is what you miss present only for my effective diffusivity coefficient formula or is it already present with Kays correlation? I do not propose any structural change in the diffusive term of the energy equation so I do not understand where or how there could be an issue that would not be pre-existing.

# Reviewer report: Reviewer response for version 1

**DOI:** 10.21956/openreseurope.16612.r32061

**Published:** 2023-06-08

**Authors:** Davide Modesti

**Affiliations:** 1 Delft University of Technology, Mekelweg, Delft, The Netherlands

The manuscript introduces the concept of "flow Prandtl number", namely a universal constant that does not depend on the fluid. This concept is introduced theoretically but then it is not verified using available experimental or numerical data, which is a major shortcoming of this work.

 I believe that the manuscript has the following shortcomings:

1. I don't understand why we need to introduce a flow Prandtl number. Isn't it enough to say that the turbulent Prandtl number is independent from the molecular Pr?
2. My main concern is that conclusions are not supported by results. After the derivation of the expression for the flow Prandtl number, I would have expected a validation of the theory using available numerical data. There are many data on this topic. For instance, we have recently generated a comprehensive dataset of turbulent plane channel flow spanning Prandtl numbers between 0.0025-4, which also covers the low Prandtl number regime of this manuscript. The manuscript is available on JFM (Pirozzoli and Modesti, 2023 ^1^) and the data are also available online at http://newton.dma.uniroma1.it/. 
3. The other major concern that I have is on the effective diffusivity in equation (4). The canonical definition of thermal diffusivity (equation 2) is not arbitrary, but it stems from the mean temperature equation upon introducing the eddy diffusivity hypothesis. How does equation (4) comply with the mean temperature balance?
4. The new formulation should be validated against reference numerical data and other state of the art formulas for the turbulent Prandtl number, such as the one by Cebeci (1973 ^2^), or the variant proposed by Na and Habib (1973 ^3^), or the formula by Kays *et al.* (1980 ^4^).
5. In the text of the manuscript I see reference to figures, but I cannot visualize any figure.

Is the study design appropriate and does the work have academic merit?

Partly

Is the work clearly and accurately presented and does it cite the current literature?

Partly

If applicable, is the statistical analysis and its interpretation appropriate?

Not applicable

Are all the source data underlying the results available to ensure full reproducibility?

No source data required

Are the conclusions drawn adequately supported by the results?

No

Are sufficient details of methods and analysis provided to allow replication by others?

Yes

Reviewer Expertise:

wall turbulence; turbulent flows; compressible flows; forced thermal convection; heat transfer

I confirm that I have read this submission and believe that I have an appropriate level of expertise to state that I do not consider it to be of an acceptable scientific standard, for reasons outlined above.

## References

[1] : Direct numerical simulation of one-sided forced thermal convection in plane channels. *Journal of Fluid Mechanics* .2023;957: 10.1017/jfm.2023.104 10.1017/jfm.2023.104

[2] : A Model for Eddy Conductivity and Turbulent Prandtl Number. *Journal of Heat Transfer* .1973;95(2) : 10.1115/1.3450031 227-234 10.1115/1.3450031

[3] : Heat transfer in turbulent pipe flow based on a new mixing length model. *Applied Scientific Research* .1973;28(1) : 10.1007/BF00413075 302-314 10.1007/BF00413075

[4] : Convective Heat and Mass Transfer. *McGraw-Hill, New York* .1980;

# Response

**Published:** 2023-06-21

**Authors:** Vincent MOREAU

**Affiliations:** BCISP-QC, CRS4, Pula (CA), Italy

The reviewer text is indicated in italics.

*The manuscript introduces the concept of "flow Prandtl number", namely a universal constant that does not depend on the fluid.*

 That is correct.

*This concept is introduced theoretically but then it is not verified using available experimental or numerical data, which is a major shortcoming of this work.*

 The concept is indeed first introduced theoretically together with the necessary notation. The notation has been clarified giving proper names to α _0_ and ν _0_. The subscript 0 is not very satisfying but is raised necessary for consistency with the definition of Pr _0_ for which it makes sense. For a comparison with published data it is necessary to look at the consequences of the hypothesis on Pr _t_ expressed in terms of the eddy viscosity, eq. 13 which is the most practical form for numerical implementation but not for further understanding of its meaning. Before proceeding with the validation, eq. 13 is rewritten as eq. 15 to make appear clearly the near constant aspect perturbed by the “natural” small parameter nu/nu _t_.

 The concept is verified from experimental data at the very beginning of the Discussion section. In effect, eq.17 (first Kays correlation in ref.2) is retrieved by substantiation of eq.16 for Pr=0.025. As in turn eq. 16 is a first order approximation of eq. 15, put in this form for this purpose. From what I have extensively seen and read, for liquid metals, Kays’ correlation is the ultimate comparison to overcome for more elaborated turbulence thermal models, such as 3 or 4 eq. models.

 So, I see that my formula is substantially identical, up to a second order term to Kays’ correlation and I sincerely think that it is more than enough for a validation when dealing with liquid metals which is the main focus of this brief report. Besides,  my derivation gives better insight on the range of validity of the Kays correlation. This can help users avoid using it for about unit Pr _t_. As easily seen from formula 13 or 15, when Pr=Pr_0 then Pr_t becomes constant which is the choice made by the main CFD code providers with their extensive suite of validation. I have seen the constant taken either at 0.85 or 0.9. It means that there is already there a non negligible uncertainty. This is also why I do not think that going above the first order approximation for validation makes sense.

*I believe that the manuscript has the following shortcomings:*

*I don't understand why we need to introduce a flow Prandtl number. Isn't it enough to say that the turbulent Prandtl number is independent from the molecular Pr?*

 This is all the point of this brief report. While the hypothesis you state is usually sufficient for engineering application it is no more when dealing with liquid metals. In this case, the region where nu and nu_t are of similar order is much wider that usual and simple summation leads to an overestimation of the effective heat transfer. Would it have been an underestimation, things would have been easier because a constant Pr_t would have provided a slightly conservative solution. Modifying the constant value can be done a posteriori but is not satisfying for prediction capabilities. Besides, we commercial CFD users have the pretention not only to give one or two global parameters but also to give better global fields description. So, it is necessary at least, and still waiting for better, to use Kays’ correlation. Problem is that there are two such correlations and authors have also interpolated between them. We are still stuck with the difficulty to reach predictability of the results.

 By giving a theoretical foundation to the Kays correlation which address the issue of the dependence of Pr_t on Pr, I believe this brief node can give a useful contribution.

**

*My main concern is that conclusions are not supported by results. After the derivation of the expression for the flow Prandtl number, I would have expected a validation of the theory using available numerical data. There are many data on this topic. For instance, we have recently generated a comprehensive dataset of turbulent plane channel flow spanning Prandtl numbers between 0.0025-4, which also covers the low Prandtl number regime of this manuscript. The manuscript is available on JFM (Pirozzoli and Modesti, 20231) and the data are also available online at http://newton.dma.uniroma1.it/.*

 This point has been abundantly answered before. The validation comes directly from the extensively validated Kays’ correlation from the one hand and from the constant value when Pr=Pr_0 fro the other hand.

**

*The other major concern that I have is on the effective diffusivity in equation (4). The canonical definition of thermal diffusivity (equation 2) is not arbitrary, but it stems from the mean temperature equation upon introducing the eddy diffusivity hypothesis. How does equation (4) comply with the mean temperature balance?*

 I have named α _0_ and ν _0_ to clarify the procedure. The description with (α _0_, ν _0_) and (α _t_, ν _t_) are completely equivalent and can be retrieved one another. The first couple comes from physical/stochastic consideration and the second couple from the averaging procedure of the NS equations. They describe the same thing with a different couple of variables. It is only a reversible change of variable from eq. 4 to eq. 9. You can also see (alpha_0, nu_0) as intermediate variables aimed at a simple definition of Pr_0, somewhat like imaginary numbers have been used for so many years as a trick to factorize 3dt and 4 ^th^ order polynomials.

**

*The new formulation should be validated against reference numerical data and other state of the art formulas for the turbulent Prandtl number, such as the one by Cebeci (19732), or the variant proposed by Na and Habib (19733), or the formula by Kays et al. (19804).*

 The new validation is indeed validated against the formula by Kays but taking his much more recent, even if already quite old, comprehensive paper of 1994, see. Ref. 2, which is precisely the core of the validation.

**

*In the text of the manuscript I see reference to figures, but I cannot visualize any figure.*

 The reference to a figure is given in the penultimate paragraph of the Discussion section. It is written “see figure 5 in 7” where 7 is the number for the reference and in effect the figure is not in the brief report. There is a very large number of figures in the referenced document and I thought it would ease the reader who would like to look at the source and not only rely on the description following the reference.

 This reference is particularly important because before its publication I could not find, probably my bad, any ν _t_ profile usable for interpretation and/or reference. I indeed postponed the submission of this brief report until the reference 7 is at least theoretically publicly available.

 My first brief report submission was much more concise, but at the editorial level I was asked to develop some arguments and then new requests arise about the new development in something that looked like and endless loop. Most of the Discussion section arises from this interaction. I had to put a stop to it, because I want this brief report to remain a brief report. It is also why I do not want to add any graph or picture. That would restart the endless loop without adding substance. If one point must be kept after the validation part, it is that it would be very easy to discard my finding, independently of its validity, by testing it against some of the popular 2-eq models, just because my formula relies on the ν _t_ profile and this ν _t_ profile is very badly reproduced, as shown in ref.7. Unfortunately, even if valid, the new formula do not lead directly to much simulation result improvement. However, it suggests to work on the existing 2-equations turbulence models so that they can procure valid ν _t_ profiles and not only the correct pressure loss.

 As a final word, please also look at the “Amendment from Version 1”. Looking at the brief report after a few month, let’s say with refreshed eyes, I could see and correct many imprecisions and/or incorrect wording. I hope the version 2 of the document is now more clear and easier to understand.

# Reviewer report: Reviewer response for version 1

**DOI:** 10.21956/openreseurope.16612.r32057

**Published:** 2023-05-30

**Authors:** Giacomo Barbi, Lucia Sirotti

**Affiliations:** 1 Università di Bologna, Bologna, Italy; 2 Department of Industrial Engineering, University of Bologna, Bologna, Emilia-Romagna, Italy

The present article deals with the analysis of an explicit formula for the turbulent Prandtl number (Pr _t_) evaluation. The use of a square additivity approach leads to a new formulation that is coherent with other well-known expressions from turbulence modeling literature. The author extends a previous result, improving upon it by avoiding the degeneration of Pr _t_ to zero or infinity. Moreover, the author's formula can be related to the Kays correlation by considering a first-order development of the full new expression.

 Overall, this work is interesting for thermal turbulence modeling, and the manuscript is well-organized. However, there are a few issues that need clarification:

- Since the meaning of Pr _0 _can be seen as the asymptotic value of Pr _t_, it is suggested to underline the meaning of α _0_ and ν _0_. It should be noted that these properties are introduced with the same description as α _t_ and ν _t_, representing properties of the flow rather than the fluid.
- In formula 15 a bracket is missing.
- It is recommended, if possible, to include a plot depicting the behavior of the new formulation of Pr _t_. The plot should consider a comparison with the Kays formula and highlight the different situations described in the first paragraphs of the Discussion section.

Is the study design appropriate and does the work have academic merit?

Yes

Is the work clearly and accurately presented and does it cite the current literature?

Yes

If applicable, is the statistical analysis and its interpretation appropriate?

Not applicable

Are all the source data underlying the results available to ensure full reproducibility?

No source data required

Are the conclusions drawn adequately supported by the results?

Partly

Are sufficient details of methods and analysis provided to allow replication by others?

Yes

Reviewer Expertise:

Turbulence modeling of low Prandtl number fluid. Finite element implementation of thermal turbulence models for liquid metal.

We confirm that we have read this submission and believe that we have an appropriate level of expertise to confirm that it is of an acceptable scientific standard.

# Response

**Published:** 2023-06-06

**Authors:** Vincent MOREAU

**Affiliations:** BCISP-QC, CRS4, Pula (CA), Italy

Thank you very much for your fast and accurate review. About the 3 issues raised:

1. You are totally right. I struggled a lot to make things clear without so much success. What I intend to do for improvement is to names the quantities more accurately:
  - remove "turbulent" after eq. 4
  - add "the flow viscosity nu_0" before eq. 6
  - add "the flow diffusivity alpha_0" before eq. 7
2. In formula 15, the last bracked must indeed be removed
3. I would definitively prefer not to add a plot. This is a brief report and is already too much extended for my taste, as I had to compose with the editorial team. What you recommend, while surely of interest, would bring this brief report outside of its domain of definition.

I will wait for another review before making an update. Thank you again.
